# Antisense oligodeoxynucleotides targeting ATM strengthen apoptosis of laryngeal squamous cell carcinoma grown in nude mice

**DOI:** 10.1186/1756-9966-30-43

**Published:** 2011-04-17

**Authors:** Jun Feng, Jian Zou, Li Li, Yongsheng Zhao, Shixi Liu

**Affiliations:** 1 From the Department of Otolaryngology-Head and Neck Surgery, West China hospital of Sichuan University, Chengdu, PR China 610041; 2 Department of pathobiology, North Sichuan Medical College, Nanchong, PR China 637000; 3 Department of cardiothoracia Surgery, West China hospital of Sichuan University, Chengdu, PR China 610041; 4 Sichuan University and working in the Affiliated Hospital of North Sichuan Medical College

**Keywords:** ATM, Antisense oligodeoxynucleotides, apoptosis, squamous cell carcinoma

## Abstract

**Background:**

To conserve laryngeal function and elevate living quality of laryngeal squamous cell carcinoma (LSCC) patients, we designed antisense oligodeoxynucleotides (AS-ODNs) to reduce expression of ATM and to enhance the apoptosis of hep-2 (Human epidermoid laryngeal carcinoma) cells to radiation in vitro and in vivo.

**Methods:**

The expression of ATM mRNA and protein in hep-2 cells were examined by real-time quantitative PCR and western blotting respectively. Clonogenic survival assay was carried out to detect the survival ability of hep-2 cells after irradiation, and analyzed the cell apoptosis by flow cytometry. The volume of solid tumors was measured, while TUNEL assay and western blotting used to analyze cell apoptosis and protein expression after irradiation.

**Results:**

The relative ATM mRNA and protein expression in hep-2 cells treated with ATM AS-ODNs were decreased to 11.03 ± 2.51% and 48.14 ± 5.53% of that in untreated cells respectively (P <0.05). After irradiation, the survival fraction (SF) of cells treated with ATM AS-ODNs was lower than that of other groups at the same dose of radiation (P < 0.05). The inhibition rate in hep-2 cells solid tumor exposed to X-ray alone was 5.95 ± 4.52%, while it was 34.28 ± 2.43% in the group which irradiated in combination with the treatment of ATM AS-ODNs (P < 0.05). The apoptotic index for the group irradiated in combination with ATM AS-ODNs injection was 17.12 ± 4.2%, which was significantly higher than that of others (P < 0.05).

**Conclusion:**

AS-ODNs of ATM reduce ATM expression and enhance hep-2 cells apoptosis to radiation in vitro and in vivo.

## Introduction

With advanced technique development in treatments of LSCC, radiotherapy is superior in its ability to conserve function in the treatment of initial laryngeal squamous cell carcinoma (LSCC). However, because of laryngeal cancer radiation resistance, which result in the low effectiveness and high recurrence when treated with radiotherapy alone [1,2]. So it is important significance to improve the LSCC radiosensitivity. Hep-2 cells, or laryngeal squamous cell carcinoma cell lines, are helpful in studying the biological behavior of LSCC. In the latest study, Hep-2 cells were found to be resistant to radiotherapy [3]. Ataxia-telangiectasia (A-T) is characterized by impaired recognition and repair of DNA damage and increased sensitivity to ionizing radiation (IR) in cancer, and neurodegeneration [4]. The cytotoxicity of ionizing radiation is mainly mediated through the generation of DNA-double strand break (DSB) as evidenced by the pronounced radiosensitivity of cells and organisms defective in the machinery of DSB repair[5-7]. Thus, restraint of DSB repair reveals a mechanism to enhance the cytotoxicity of IR in tumour cells. ATM (ataxia telangiectasia mutated) is a key protein responsible for arresting the cell cycle in response to DNA damage and has a role in genetic stability and cancer susceptibility [8-10]. ATM protects the integrity of the genome at different levels: (1) it mediates arrest of the cell cycle at G_1_/S, S, and G_2_/M to prevent the processing of damaged DNA; (2) it activates DNA-repair pathways; and (3) it induces apoptosis if the DNA damage is so detrimental that normal cell function can no longer be rescued [11-15]. Zou and colleagues have shown that antisense inhibition of ATM gene enhances the radiosensitivity of head and neck squamous cell carcinoma in mice [16,17]. Sak A reported that the kinase activity of DNA-PKcs could be specifically inhibited by As-ODNs and resulted in marked inhibition of DNA-Dsb rejoining and radiosensitization of human non-small cell lung cancer (NSCLC) cell line [18]. Leonard CE's study showed that the Paclitaxel could enhance the radiosensitivity of squamous carcinoma cell line of the head and neck in vitro [19]. However, there were no reports about the antisense oligodeoxynucleotides of ATM strengthening radio-induced apoptosis of laryngeal squamous cell carcinoma grown in nude mice. Therefore, we designed to study whether reduction of ATM expression after antisense oligodeoxynucleotides (AS-ODNs) treatment would result in enhanced radio-induced apoptosis of Hep-2 cells from BALB/c-nu/nu mice.

## Methods

### Reagents

Lipofectamine 2000, Opti-MEM I medium and Trizol kit were bought from Invitrogen Company (Carlsbad, CA, USA), and anti-GAPDH Monoclonal Antibody from SAB (Beijing, China). SYBR ExScript RT-PCR Kit, SYBR Green Master Mix, AnnexinV-FITC-PI, RPMI-1640 media and 10% heat-inactivated fetal bovine serum (FBS) were purchased from Takara Biotechnology Company (Dalian, China). ATM monoclonal antibody was bought from Santa Cruz Biotechnology (Santa Cruz, CA, USA). BCIP/NBT alkaline phosphatase substrate kit IV was purchased from Vector laboratories (Burlingame, CA, USA). TUNEL apoptosis detection kit was bought from Roche Company (Shanghai, China).

### Cell lines and mice

Hep-2 cell line was obtained from the laboratory of Head and Neck at Sichuan University. The cells were maintained in RPMI-1640 medium, supplemented with 10% heat-inactivated fetal bovine serum, 100 μg/mL streptomycin, and 100 U/mL penicillin G in a humidified atmosphere of 5% CO_2 _and 95% air at 37°C. Female BALB/c-nu/nu mice, aged 3-4 weeks, weighing 18-22 g, were obtained from the animal centre of West China Medical School and were maintained in the animal facility at West China Medical School, Sichuan University in accordance with nation's related regulations and animal welfare requirements.

### Synthesis of oligodeoxynucleotides (ODNs) and selection of target sequences

AS-ODNS, sense (Sen) and mismatch (Mis) ODNs were synthesized by Shanghai Sangon Biological Engineering Technology & Services (Shanghai, China). The sequences were as follows: AS (5'-GTACTAGACTCATGGTTCACAATTT-3'); Sen (5'-AAATTGTGAACCATGAGTCTAGTAC-3') and Mis (5'-AAAATGTAAACCATAAGTCTAGAAC-3'). All the ODNs were chemically modified to phosphorothioate ODNs by substituting the oxygen molecules of the phosphate backbone with sulfur.

### Transfection of ODNs in Hep-2 cells

Hep-2 cells at a density of 2 × 105 cells/ml were plated in 6-cell plates for overnight incubation. Cells were maintained in RPMI-1640 medium supplemented with 10% FBS at 37°C and 5% CO2. After grew to 70-80% confluent, cells were replenished with incomplete RPMI-1640 medium, then treated with ATM AS-ODNs, ATM Sen-ODNs and Mis-ODNs. The procedures were as follows: 0.8 ug of ATM AS-ODNs, Sen-ODNs, Mis-ODNs and 2 mg/ml Lipofectamine 2000 were added to Opti-MEM I medium separately, and incubated for 5 min at room temperature. Then liposome and ODNs were mixed and incubated at room temperature for 20 min. Hep-2 cells were washed again with Opti-MEM I medium before transfection. The liposome ODNs complexes were carefully plated on the cells, and incubated at 37°C, 5% CO2. After 6 hours transfected cells were washed twice with PBS. With the medium replaced with fresh RPMI-1640 medium supplemented with 10% FBS, the cells were incubated at 37°C overnight. A second ODNs incubation was performed before cells were exposed to radiation.

### Real-time quantitative PCR analysis

According to the manufacturer's recommendations total RNAs were extracted from cultured Hep-2 cells using Trizol reagent. One-step RT-PCR was performed in LightCycler-RNA Amplification Kit SYBR Green I. ATM was amplified with the sense primer: (5'-GACCGTGGAGAAGTAGAATCAATGG-3' and the anti-sense primer: 5'-GGCTCTCTCCAGGTTCGTTTGC-3'). GAPDH (sense primer: 5'-GAAGGTGAAGGTCGGAGT-3', anti-sense primer: 5'-GAAGATGGTGATGGGATTTC-3') was used as a housekeeping gene, in order to normalize the expression of target genes. The reaction mix consisted of 6 mmol/L MgCl_2_, 0.4 μl LightCycler-RT-PCR Enzyme Mix and 4 μl LightCycler-RT-PCR Reaction Mix SYBR Green I. All oligonucleotide primers were designed and synthesized by Sangon (Shanghai, China). All primers used were at 0.5 μmol/L final concentration. The thermal cycling conditions were as follows: 10 min at 55°C for reverse transcription, 30 seconds at 95°C for pre-denaturation, 42 cycles for 1 second at 95°C for denaturation, 10 seconds at 62°C for annealing and finally, 13 seconds at 72°C for elongation. At the end of each cycle, the fluorescence emitted by the SYBR Green I was measured. After completion of the cycling process, samples were immediately subjected to a temperature ramp for melting curve analysis. The relative abundance of target mRNA in each sample was calculated using the formula suggested by Muller et al[20] which is given by 2^-(IL-8 Threshold Cycle)^/2^-(β-actin Threshold Cycle) ^× 10^6 ^.

### Western blot analysis

Total proteins extracted from Hep-2 cells were separated on 10% or 15% DS-polyacrylamide gels. The procedure was briefly described as following: 40 micrograms of cell extract was separated electrophoretically using sodium dodecyl sulfate-polyacrylamide gel electrophoresis gel and transferred to nitrocellulose membranes. The membrane was blocked with 3% milk powder in nonfat milk in phosphate-buffered saline (PBS) at room temperature for 6-8 hours, washed with PBST (PBS containing 0.1% Tween-20) for 10 min three times. The blot was incubated overnight at 4°C with rabbit anti-ATM monoclonal antibody per mL in PBS containing 2.5% nonfat milk, 2.5% bovine serum albumin (BSA), and 0.1% Tween 20. The membrane was washed with PBS containing 0.1% Tween 20 for 15 min (×4). The membrane was incubated with alkaline phosphatase-labeled anti-mouse IgG antibody in TBS containing 1% milk powder at room temperature for 1 hour and washed again with TBS for 15 min (×1), then 5 min (×4). Using the BCIP/NBT alkaline phosphatases substrate kit IV, the membrane was briefly visualized. Reactive bands were scanned by Gel Doc 1000 (Bio-Rad). The experiment was repeated three times.

### Irradiation

GWGP-60 Precise radiation system (Beijing, China) was used to irradiate cells and solid tumor. X-ray irradiation was carried out at room temperature at a dose rate of 200 cGy/min and equipped with an external 0.5-mm copper filter.

### Clonogenic survival assay

Preliminary studies were conducted to optimize the number of cells plated in clonogenic assays, aiming at 100 colonies per well. The Hep-2 cells were seeded in triplicate at limiting dilutions in 6-well plates for about 24 hours in RPMI-1640 medium supplemented with 10% FBS. Then the cells were transfected with ATM AS-ODNs, ATM Sen-ODNs and Mis-ODNs respectively. About 18 hours after transfection, they were irradiated simultaneously with different doses of X-ray radiation (0, 2, 4, 6, and 8 Gy) respectively. The medium was replaced with a fresh one 24 hours after irradiation. Colonies were fixed and stained with 0.5% crystal violet, and the number of colonies containing at least 50 cells, as examined by microscopy, was recorded 3 to 7 days later. In each irradiation dose group, surviving fraction (SF) of cells was calculated as plating efficiency of the irradiated cells divided by the plating efficiency of untreated samples.

### Apoptosis analyzed by flow cytometry

After 48 hours exposed to 4 Gy radiation, Hep-2 cells were harvested, and centrifuged at 1500 rpm for 2 min. Then cells were washed with PBS twice, and fixed in ice-cold 70% ethanol at 4°C overnight. After rinsing 1 × 105-1 × 106 cells with 1× Binding Buffer, the cells were reharvested and resuspended in 200 μl of 1× Binding Buffer. 5 μl of Annexin V and 10 μl of Propidium Iodide (PI) were added in cells incubating at room temperature for 15 min in the dark. Cell apoptotic rate were analyzed by flow cytometry (Elite ESP, BeckmanCoulter, USA).

### Animal experiment

Female BALB/c-nu/nu mice were used to investigate the effect of ATM AS-ODNs on radio-induced apoptosis of Hep-2 cells solid tumor. All surgical procedures and care administered to the animals were in accordance with institutional guidelines. Animal surgeries and radiotherapy were performed under general anesthesia, 50 mg/kg ip injection of pentobarbital sodium. About 1 × 10^5 ^Hep-2 cells were subcutaneously inoculated in submental space of the mice. Tumor growth rates were determined by measuring two orthogonal dimensional diameters of each tumor thrice a week. Tumor volumes were calculated according to the formula V = π/6 × a^2 ^× b, where a is the short axis, and b the long axis. When tumors reached an average volume of about 200 mm3, the tumor-bearing BALB/c-nu/nu mice were divided into four groups assigned 8 nude mice in each group: (a) control group, no treatment; (b) ATM AS-ODNs group, tumors were treated with ATM AS-ODNs alone but not exposed to irradiation for each time; (c) irradiation group, tumors were exposed to X-ray of 2 Gy alone for each time; and (d) combination group, 2.5 mg/kg of ATM AS-ODNs was injected into the solid tumor the day before X-ray exposure, another dosage of ATM AS-ODNs was injected right before exposure to 2 Gy of X-ray for each time. The same treatment for each group was repeated 3 times (the interval time was 5 days). BALB/c-nu/nu mice were killed 3 weeks later. The ATM protein expression of the tumor in the different groups was analyzed by western blot using the procedures described as above. The tumor inhibition rate was calculated using the following formula: (1-average tumor volume of experimental group/average tumor volume of control group) ×100%.

### TUNEL assay

TUNEL (Terminal deoxynucleotidyltransferase-mediated dUTPdigoxigenin nick-end-labeling) staining of tumor sections was performed using an in situ apoptosis detection kit (Roche, Shanghai, China) according to the manufacture's protocol. The total number of apoptotic cells in 10 randomly selected fields was counted. The apoptotic index (AI) was calculated as the percentage of positive staining cells, namely AI = number of apoptotic cells × 100/total number of nucleated cells.

### Statistics

Results were expressed as mean ± standard deviation (SD) and were analyzed with a one-way ANOVA and SPSS18.0 software package used to perform statistical analysis. A value of P < 0.05 was considered significant between the experimental groups compared with other groups.

## Results

### Expression of ATM in ATM AS-ODNs transfected Hep-2 cells

**Figure 1:**
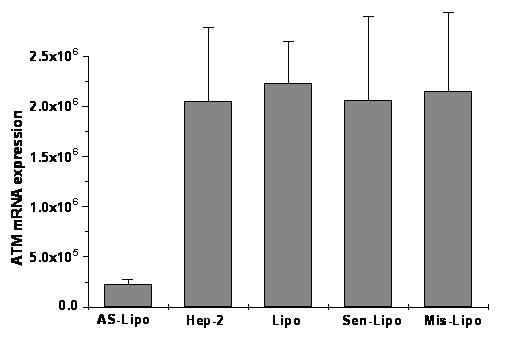
**Real-time quantitative PCR analysis of ATM mRNA expression**. Liposome formulations of ATM AS-ODNs decreased expression of ATM mRNA was notably lower than that of other groups. There are no significant differences among liposome-treated group (Lipo), Sen-ODNs (Sen-Lipo) treated group and Mis-ODNs (Mis-Lipo) treated group (P > 0.05). P < 0.05, AS-ODNs (AS-Lipo) treated group compared with other groups.

**Figure 2:**
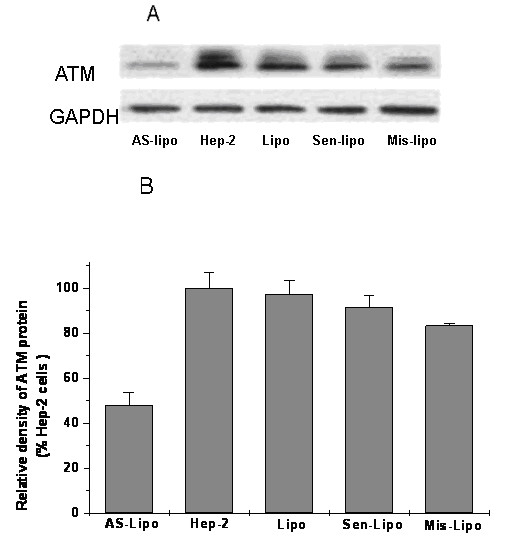
**A Effect of ATM AS-ODNs on the expression of ATM protein in vitro**. (A) Liposome formulations of ATM AS-ODNs dramatically reduced the expression of ATM protein compared with other groups. (B) There are no significant differences among liposome-treated group (Lipo), Sen-ODNs (Sen-Lipo) treated group and Mis-ODNs (Mis-Lipo) treated group (P > 0.05), while the group treated with ATM AS-ODNs notably different compared with other groups (**P <0.05).

To analyze the expression of ATM in mRNA and protein level in Hep-2 cells, real-time fluorescent quantitative PCR and western blot assay were used respectively. It is evident that there were no significant differences among the groups treated with liposome alone, with Sen-ODNs and with Mis-ODNs after 72 hours treatment (P > 0.05; Figure 1). However when incubated with liposome formulations of ATM AS-ODNs, the relative ATM mRNA expression was only about 11.03 ± 2.51% to the untreated Hep-2 cells, which showed a significantly reduced expression of ATM mRNA (P < 0.05;Figure 1). As shown in Figure 2, ATM protein expression was also significantly reduced by ATM AS-ODNs compared with Sen-ODNs and Mis-ODNs after 72 hours treatment (Figure 2A). The relative ATM protein expression of Hep-2 cells treated with ATM AS-ODNs was only about 48.14 ± 5.53% to the untreated cells (P < 0.05; Figure 2B). But there was no significant difference among the group treated with liposome alone, the group treated with Sen-ODNs, the group treated with Mis-ODNs and the group of control untreated Hep-2 cells (P > 0.05; Figure 2B).

### ATM AS-ODNs on clonogenic survival ability of Hep-2 cells after irradiation

**Figure 3:**
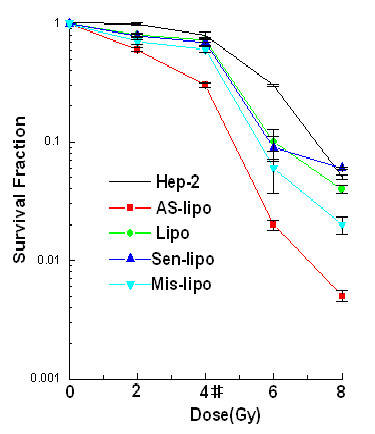
**Survival curves for Hep-2 cells after irradiation**. Survival fractions at each dose point were normalized to untreated cells. * P < 0.05, the mean of SF4 in the cells transfected with ATM AS-ODNs was significantly lower than that of other cells.

Cloning efficiency, P <0.05, was declined in cells transfected with ATM AS-ODNs compared with untreated cells or cells treated with control at the identical radioactive dose (Figure 3). After 4 Gy radiation, the survival fraction (SF4) revealed the cellular radio-induced apoptosis. The SF4 of cells transfected with ATM AS-ODNs was 31.3 ± 5.1%, notably lower than that of other cells, which indicated a definite increase in the radio-induced apoptosis (P < 0.05; Figure 3). In clonogenic survival ability, there were no significant differences compared with other groups (P > 0.05; Figure 3).

### Apoptosis of Hep-2 cells after irradiation in vitro

**Figure 4:**
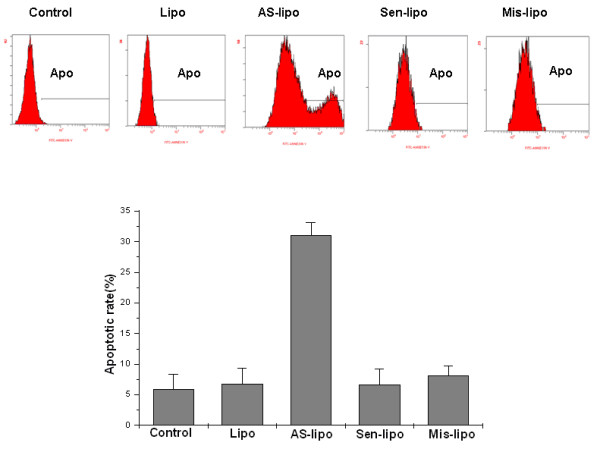
**The apoptotic rate of Hep-2 cells after 4 Gy irradiation**. P < 0.05, the apoptotic rate (Apo) in ATM AS-ODNs transfected cells compared with that in Sen-ODNs, Mis-ODNs and Lipofectamine transfected cells after 4 Gy irradiation. * P > 0.05, no significant differences among Sen-ODNs, Mis-ODNs, Lipo and control groups.

After 4 Gy irradiation, the apoptotic rate in ATM AS-ODNs transfected cells was 30.7 ± 1.31%, which was higher than that in Sen-ODNs and Mis-ODNs transfected cells (P < 0.05; Figure 4).

### Inhibitory effect of ATM AS-ODNs on tumor growth in vivo after irradiation

**Figure 5:**
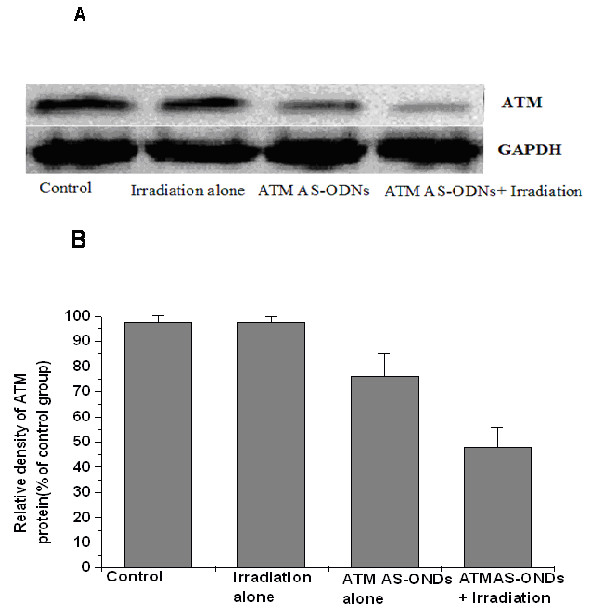
**Effect of ATM AS-ODNs on the ATM protein expression in vivo**. (A) In the group treated with ATM AS-ODNs alone (ATM AS-ODNs treated alone) and the group irradiated in combination with ATM AS-ODNs (ATM AS-ODNs + irradiation), the expression of ATM protein were decreased. (B) * P < 0.05, compared with the group irradiated in combination with ATM AS-ODNs and the group irradiated alone.

The homologous ATM protein expression were only 76.84 ± 3.12% and 48.19 ± 3.98% to the untreated group respectively in the group treated with ATM AS-ODNs alone and the group irradiated in combination with the treatment of ATM AS-ODNs (P < 0.05; Figure 5). Tumor growth of the mice in four groups was shown in Figure 5. The inhibition rate in Hep-2 cells solid tumor treated in X-ray alone was 5.95 ± 4.52%, while it was 34.28 ± 2.43% in solid tumor irradiated in combination with the treatment of ATM AS-ODNs at the experimental endpoint(P < 0.05;Figure 5).

**Figure 6:**
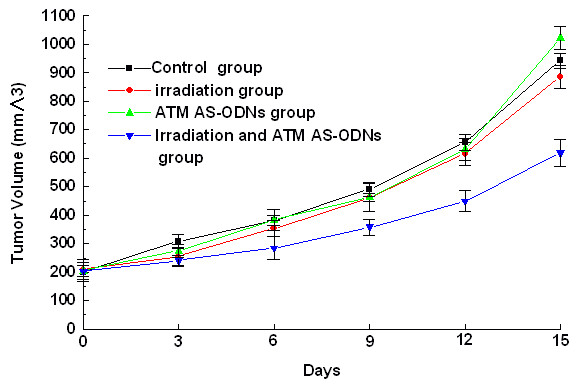
**Tumor growth in ATM AS-ODNs treated Hep-2 cells in BALB/c-nu/nu mice with or without irradiation**.

### Enhancement of tumor apoptosis by irradiation combined with ATM AS-ODNs treatment in vivo

**Figure 7:**
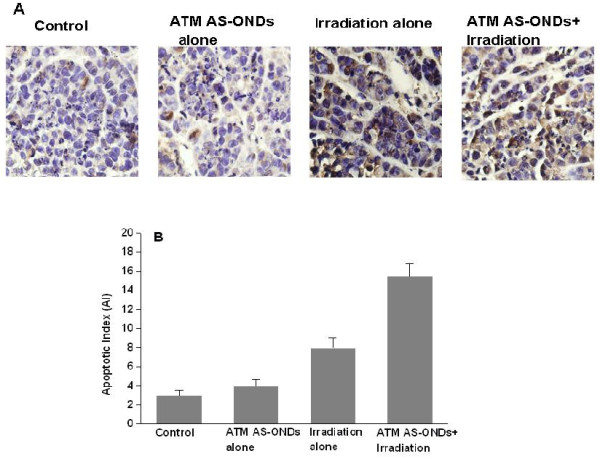
**The apoptosis of Hep-2 cells in vivo after irradiation**. (A) The detection of apoptotic cells are by TUNEL. The nuclei of apoptotic cells were stained brown as observed under light microscopy (magnification, × 400). (B) The apoptotic rate of tumor cells treated by irradiation in combination with ATM AS-ODNs was raised. * P < 0.05, the AI of tumors treated with irradiation in combination with ATM AS-ODNs compared with the untreated group and the group treated with ATM AS-ODNs alone. ** P < 0.05, compared with the other groups.

There were small numbers of apoptotic cells detected by TUNEL analysis in tumors treated with irradiation alone, while the group treated with irradiation in combination with ATM AS-ODNs was notably higher than that of irradiation alone (Figure 7A). Accordingly, the AI for mice tumors treated with irradiation in combination with ATM AS-ODNs was 17.12 ± 4.2%, significantly higher than that of the other groups (P <0.05; Figure 7B).

## Discussion

Phosphorylation of several DNA damage response proteins, including ATM, p53, can be observed in precursor stage cancers of the breast, colon, lung, skin, testes, and urinary bladder [21-23]. It may suggest that DNA damage occurs during the earliest stages of tumor development, before genomic instability and the loss of wild-type p53 function in many cancers. Raju V have demonstrated that p53 induction in response to Myc overexpression requires the ataxia-telangiectasia mutated (ATM) kinase, a major regulator of the cellular response to DNA double-strand breaks[24]. Mohammad A speculated that ATM deficiency might increase the sensitivity of leukemic blasts to the chemotherapy used during induction and after disease remission in patients with adult ALL (Acute Lymphoblastic Leukemia) [25]. Jian confirmed that Antisense inhibition of ATM gene enhanced the radiosensitivity of head and neck squamous cell carcinoma in mice. Therefore we designed the experiment to verify the hypothesis whether ATM AS-ODNs could inhibit the expression of ATM in Hep-2 cells and furthermore increase the radio-induced apoptosis in vitro and in vivo.

Here we show that transgenic expression of ATM AS-ODNs into hep-2 cells on its own induced the inhibitory expression of ATM at mRNA and protein level in hep-2 cells. We detected that expression of ATM was notably lower after cell transfection with ATM AS-ODNs than Sen-ODNs, Mis-ODNs and control ODNs, which showed that the inhibition was specific for the ATM antisense sequence. Then we studied whether the reduction of ATM expression resulted in radio-induced apoptosis enhancing in hep-2 cells. The results of clonogenic survival assay and SF4 demonstrated that the cloning efficiency and SF4 declined notably in cells transfected with ATM AS-ODNs at the same dose of radiation (P < 0.05) compared with untreated cells or cells treated with liposome, which means the increase of cell apoptosis. By flow cytometry, we found that the apoptotic rate (Apo) in ATM AS-ODNs treated cells was higher than that in Sen-ODNs and Mis-ODNs treated cells after irradiation.

In the study, we also investigated the effects of ATM AS-ODNs on the apoptotic responses to ionizing radiation in vivo. It was obvious that there were a significant difference between the tumors irradiated in combination with the treatment of ATM AS-ODNs and controlling tumor. The inhibition rate in the tumors injected with ATM AS-ODNs before exposure to X-ray was 34.28 ± 2.43%, whereas it was 5.95 ± 4.52% in tumors exposed to radiation alone (P < 0.05). The results of TUNEL assay demonstrated that the apoptotic rate of the tumors irradiated in combination with the treatment of ATM AS-ODNs was notably higher than that of control groups. The findings in vivo experiments manifested that the radio-induced apoptosis of hep-2 cells in solid tumors were enhanced by the treatment of ATM AS-ODNs, which may be related with the increased radiosensitivity and radiation-induced apoptosis. Jian and colleagues have shown that antisense oligodeoxynucleotides of ATM enhances the radiosensitivity of head and neck squamous cell carcinoma in mice [16,17]. We had demonstrated that the ATM AS-ODNs could specifically reduce the ATM expression and increase radio-induced apoptosis in hep-2 cell line. It is first reported with AS-ODNs of ATM strengthening radio-induced apoptosis of hep-2 cell line grown in nude mice.

In conclusion, radiotherapy combined with AS-ODNs could specifically reduce the ATM expression and increase radio-induced apoptosis in hep-2 cell line. This approach might have great potential for the clinical treatment of many tumors.

## Conclusion

We had demonstrated that the ATM AS-ODNs could specifically reduce the ATM expression and increase radio-induced apoptosis in hep-2 cells in vitro and in vivo in our study.

## Competing interests

The authors declare that they have no competing interests.

## Authors' contributions

JZ participated in the design of the study and performed the statistical analysis. LL carried out cell culture and flow cytometry assay, participated in the animal experiment. YZ participated in irradiation for cells and animals. SL conceived of the study, and participated in its design and coordination and helped to draft the manuscript. JF designed the study, performed the rest of the experiments and wrote the manuscript. All authors read and approved the final manuscript.
